# Suppression of auxin signalling promotes rice susceptibility to *Rice black streaked dwarf virus* infection

**DOI:** 10.1111/mpp.12814

**Published:** 2019-06-27

**Authors:** Hehong Zhang, Xiaoxiang Tan, Lulu Li, Yuqing He, Gaojie Hong, Junmin Li, Lin Lin, Ye Cheng, Fei Yan, Jianping Chen, Zongtao Sun

**Affiliations:** ^1^ The State Key Laboratory Breeding Base for Sustainable Control of Pest and Disease Institute of Plant Virology, Ningbo University Ningbo 315211 China; ^2^ Key Laboratory of Biotechnology in Plant Protection of MOA and Zhejiang Province Institute of Virology and Biotechnology, Zhejiang Academy of Agricultural Sciences Hangzhou 310021 China

**Keywords:** auxin signalling, jasmonic acid, *OsRboh*, reactive oxygen species, rice black streaked dwarf virus

## Abstract

Auxin plays a fundamental role in plant growth and development, and also influences plant defence against various pathogens. Previous studies have examined the different roles of the auxin pathway during infection by biotrophic bacteria and necrotrophic fungi. We now show that the auxin signalling pathway was markedly down‐regulated following infection of rice by *Rice black streaked dwarf virus* (RBSDV), a dsRNA virus. Repression of the auxin receptor TIR1 by a mutant overexpressing *miR393* increased rice susceptibility to RBSDV. Mutants overexpressing the auxin signalling repressors *OsIAA20* and *OsIAA31* were also more susceptible to RBSDV. The induction of jasmonic acid (JA) pathway genes in response to RBSDV was supressed in auxin signalling mutants, suggesting that activation of the JA pathway may be part of the auxin signalling‐mediated rice defence against RBSDV. More importantly, our results also revealed that *OsRboh*‐mediated reactive oxygen species levels played important roles in this defence. The results offer novel insights into the regulatory mechanisms of auxin signalling in the rice–RBSDV interaction.

## Introduction

Auxins play key roles in almost every aspect of plant growth and development, regulating many processes by changes in their concentration. Indole‐3‐acetic acid (IAA) is the major form of natural auxin in plants (Zhao, 2010) and three components are known to be involved in auxin signal transduction: IAA receptors TIR1/AFB, transcriptional repressors Aux/IAA and auxin response factors (ARF) transcription factors (Mockaitis and Estelle, 2008; Weijers and Wagner, 2016). In the absence of auxin, Aux/IAA proteins bind to the transcription factor ARFs, repressing the expression of auxin response genes. The F‐box protein TIR1/AFB is a component of the SCF^TIR1/AFB^ ubiquitination E3 complex. When the IAA concentration increases, TIR1/AFB receptors bind Aux/IAA repressor proteins and form a SCF^TIR1/AFB^–auxin–Aux/IAA complex leading to the polyubiquitination and degradation of Aux/IAA proteins. Therefore, the degradation of Aux/IAAs allows a set of ARFs to activate their target genes. This process is essential for most physiological processes regulated by auxin (Weijers and Wagner, 2016).

Besides its role in plant development, recent studies have demonstrated that auxin is also involved in plant defence (Fu and Wang, 2011; Vidhyasekaran, 2015). For example, repressing auxin signalling by the overexpression of miR393 increased resistance of *Arabidopsis* to the bacterial pathogen *Pseudomonas syringae* (Navarro *et al.*, 2006). Auxin signalling was believed to antagonize the salicylic acid (SA)‐mediated disease resistance pathway in response to bacterial invasion (Wang *et al.*, 2007). Auxin signalling plays multiple roles in plant defence against necrotrophic fungal pathogens (Llorente *et al.*, 2008; Qi *et al.*, 2012). Disruption of the auxin signalling pathway increased susceptibility to *Alternaria brassicicola*, possibly because the induction of the jasmonic acid (JA) pathway related genes was suppressed (Qi *et al.*, 2012). However, auxin signalling and transport increased the susceptibility of *Arabidopsis* to *Fusarium oxysporum* (Kidd *et al.*, 2011). Hence, the effect of the auxin pathway on plant defence is complicated, depending on the pathogen and host.

*Rice black streaked dwarf virus* (RBSDV), a member of the genus *Fijivirus*, family *Reoviridae*, is transmitted to rice, maize, barley and wheat by the small brown planthopper (*Laodelphax striatellus* Fallén, SBPH) in a persistent, circulative manner (Wei and Li, 2016). RBSDV has an icosahedral, double‐layered particle with a diameter of about 75–80 nm and a genome that consists of ten segments of double‐stranded RNA (dsRNA) (Wang *et al.*, 2003). RBSDV infection causes severe plant growth abnormalities, such as dwarfism, and darkening of leaves, symptoms that are associated with a change of hormone homeostasis (Huang *et al.*, 2018). Indeed, our previous research revealed that the jasmonic acid (JA) and brassinosteroid (BR) pathways were changed in contrasting ways in response to RBSDV infection (He *et al.*, 2017). While the JA pathway played an important role in defence against RBSDV, the BR pathway facilitated infection (Zhang *et al.*, 2019b). In addition, the abscicic acid (ABA) pathway was also shown to negatively regulate plant defence against RBSDV (Xie *et al.*, 2018).

Here, the role of auxin signalling in RBSDV infection was investigated by using various auxin signalling mutants. The results showed that auxin signalling played a positive role in rice defence against RBSDV infection. Further results demonstrated that the JA pathway and *OsRboh*‐mediated reactive oxygen species (ROS) levels contributed to this defence.

## Results

### The effect of RBSDV infection on auxin homeostasis

**Figure 1:**
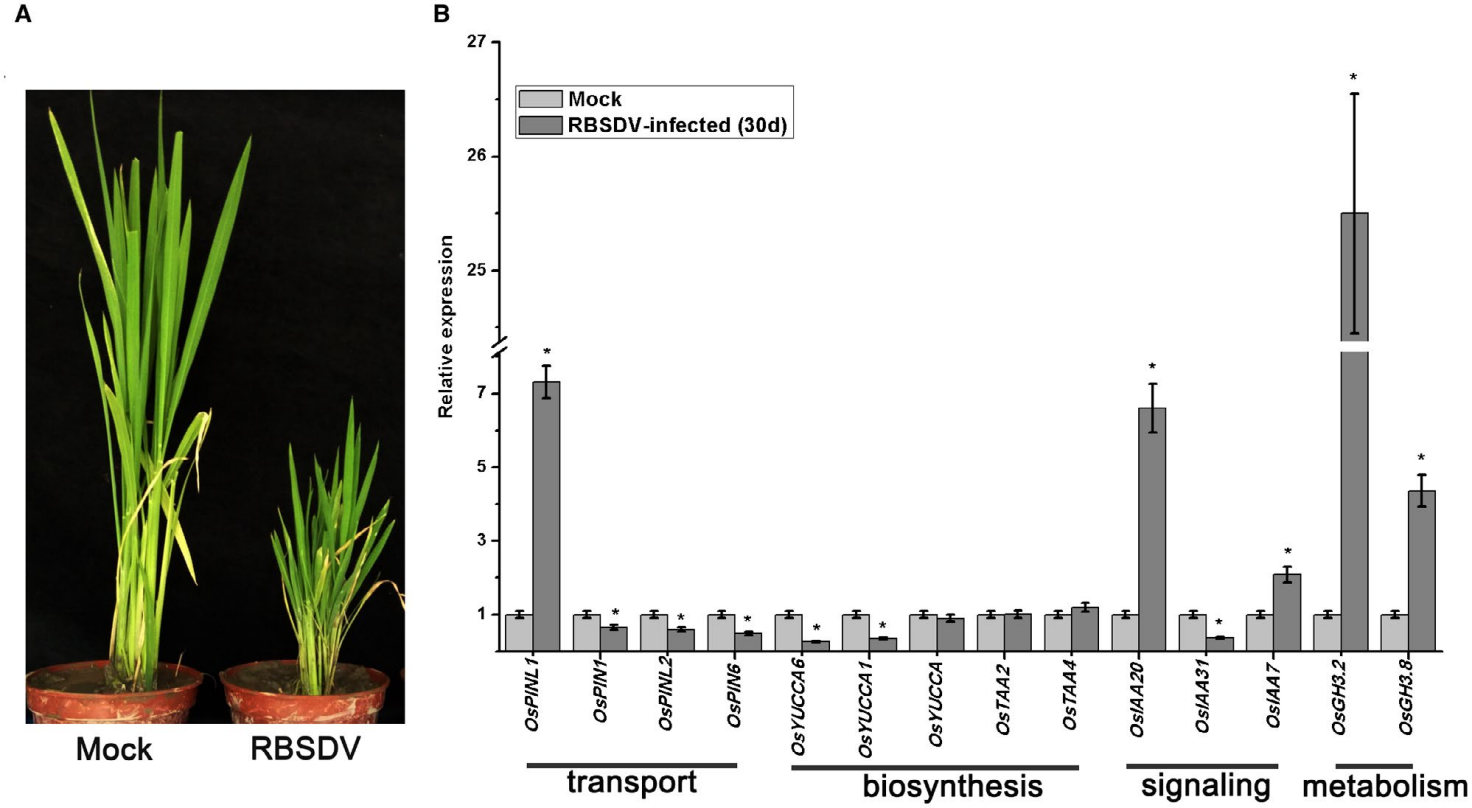
The effects of RBSDV infection on gene expression in the auxin pathway. (A) Typical symptoms of RBSDV‐infected rice compared with mock‐inoculated plant. The symptoms were observed 30 days post‐RBSDV inoculation (dpi). (B) Quantitative reverse transcription‐polymerase chain reaction (RT‐qPCR) verification of the expression of auxin pathway genes (transport, biosynthesis, signalling and metabolism) in response to RBSDV infection at 30 dpi in rice. Data are shown as relative expression levels of virus‐infected plants in comparison to the control plants. UBQ5 was used as the internal reference gene. Values are means ± SD of three biological replicates. Statistically significant differences from the control are indicated: *P ≤ 0.05.

Rice plants were severely dwarfed after RBSDV infection (Fig. 1A) and therefore the effect of RBSDV infection on the auxin pathway was investigated in rice. First, the effects of RBSDV on the expression of genes involved in auxin transport (*OsPINs*), biosynthesis (*OsYUCCAs* and *OsTAAs*), signalling (*OsIAAs*) and metabolism (*OsGH3s*) was assessed by quantitative reverse transcription‐polymerase chain reaction (RT‐qPCR) 30 days post‐infection (dpi). As shown in Fig. 1B, the auxin transport genes *OsPIN1*, *OsPINL2* and *OsPIN6* (but not *OsPINL1*) and the auxin biosynthesis genes *OsYUCCA1* and *OsYUCCA6* were significantly down‐regulated in RBSDV‐infected plants compared to the controls. The *OsGH3* family genes are reported to reduce auxin content by catalysing the conjugation of IAA to amino acids and we found that the expression of *OsGH3.2* and *OsGH3.8* was markedly increased in our RBSDV‐infected plants. Moreover, the expression of genes involved in auxin signalling (*OsIAA20*, *OsIAA31* and *OsIAA7*) was also obviously changed in response to virus infection. Interestingly, the expression pattern of *OsIAA20* and *OsIAA31* genes was converse. This might be due to the different *cis*‐element in the promoters of *OsIAA20* and *OsIAA31* (Fig. S1).

**Figure 2:**
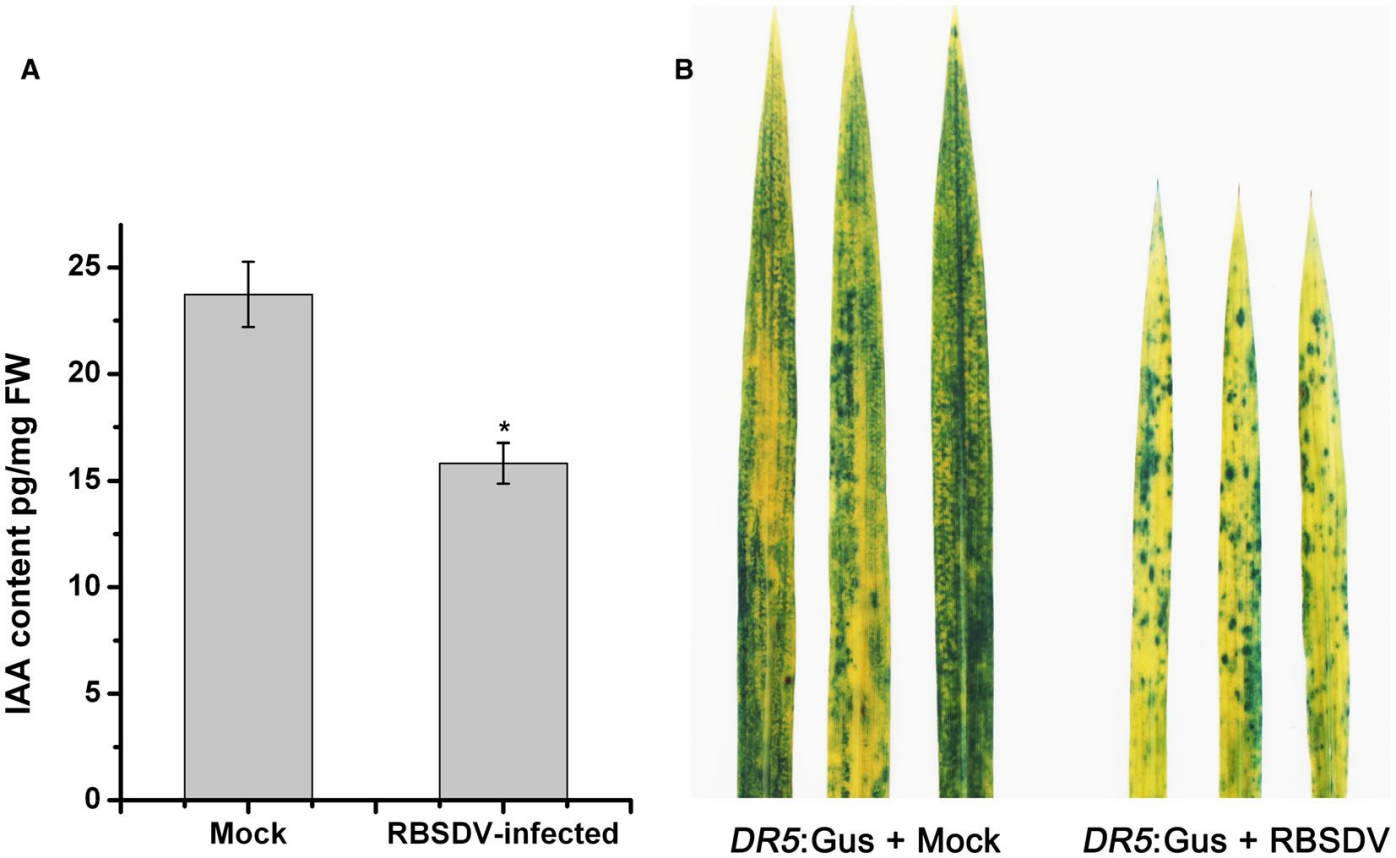
The effects of RBSDV infection on the homeostasis of the auxin pathway. (A) The IAA contents of mock‐inoculated and RBSDV‐infected rice plants at 40 days post‐inoculation (dpi). (B) Activity of the *DR5* promoter in the leaves of mock‐inoculated and RBSDV‐infected *DR5:GUS* rice transgenic plants at 40 dpi.

To determine whether the change of gene expression affected auxin homeostasis, IAA concentrations were measured in RBSDV‐infected leaves at 40 dpi by UPLC‐MS/MS (Fu *et al.*, 2012). As shown in Fig. 2A, IAA concentrations were significantly less in the infected plants (15.8 pg mg^−1^ FW) than in the mock‐inoculated controls (23.7 pg mg^−1^ FW). We next measured the IAA concentration in *DR5:GUS* transgenic plants inoculated with RBSDV. *DR5:GUS* constructs use highly active auxin‐responsive promoter elements to drive β‐glucuronidase (GUS) expression (Ulmasov *et al.*, 1997) and can therefore be used as an indicator to monitor auxin homeostasis. Consistent with the direct IAA measurements, the GUS activity in RBSDV‐infected transgenic plants was obviously reduced compared to the mock‐inoculated controls (Fig. 2B). The results all demonstrate that the expression of auxin pathway genes and auxin homeostasis was significantly affected by RBSDV infection.

### The effect of the auxin signalling pathway on RBSDV infection

**Figure 3:**
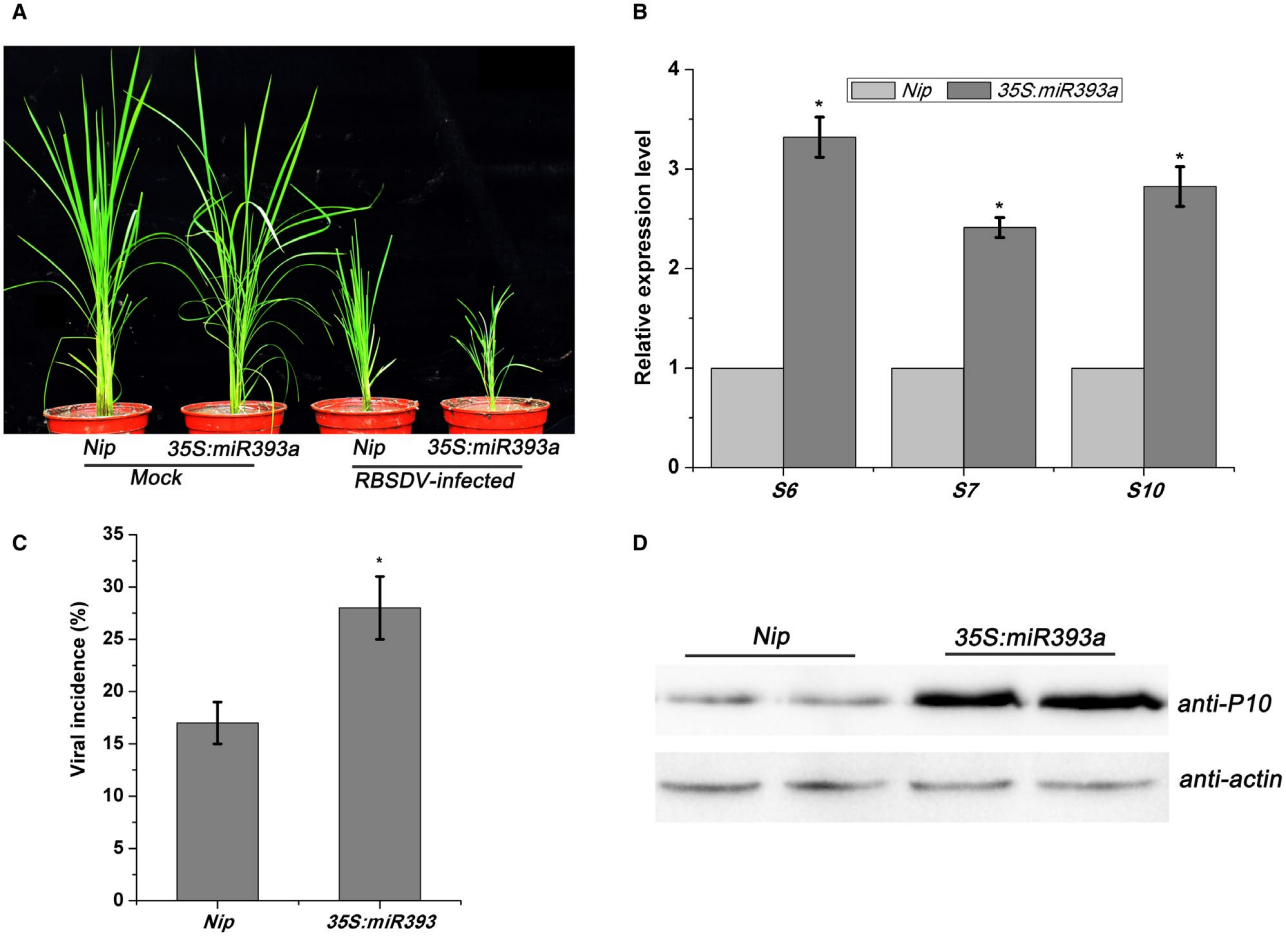
The effect of overexpression of *miR393* on RBSDV infection. (A) The symptoms on mock‐inoculated or RBSDV‐infected *Nip* and *35S:miR393a* mutant plants, respectively. The symptoms were observed at 30 days post‐inoculation (dpi). (B) The relative expression levels of RBSDV *S6*, *S7* and *S10* in RBSDV‐infected *Nip* and *35S:miR393a* mutant plants assessed by RT‐qPCR. Three biological replicates were performed. The average values from three biological replicates are shown. Error bars represent ±SD. *P ≤ 0.05. (C) Viral incidence: the numbers of healthy and diseased plants in each treatment were determined by RT‐PCR at 30 dpi. Each treatment used at least 40 seedlings and at least three biological replicates were performed. * at the top of columns indicates significant difference at *P* ≤ 0.05 (*n* ≥ 3). (D) Western blot of leaf extracts showing detection of the RBSDV P10 outer capsid protein using an anti‐P10 antibody. Actin antibody was used as an internal reference.

To explore the relationship between the auxin pathway and viral infection, we examined the susceptibility of auxin signalling mutants to RBSDV. First, we used a mutant overexpressing miR393 (*35S:miR393*) in which the expression of the auxin receptor TIR1 was significantly decreased (Bian *et al.*, 2012). 30 days after inoculating *35S:miR393* transgenic and non‐transgenic (*Nip*) plants with RBSDV using viruliferous small brown planthopper (SBPH) (or virus‐free insects for the controls), viral symptoms were examined and the concentration of viral RNA transcripts (*S6*, *S7* and *S10*) was determined. Dwarfing symptoms were more severe in the *35S:miR393* mutant than in the *Nip* controls (Fig. 3A) and the mutant plants had a significant two‐ to three‐fold increase in expression of the viral RNAs compared with *Nip* (*P* ≤ 0.05) (Fig. 3B). The viral P10 outer capsid protein levels also showed a similar increase (Fig. 3C) and there was an increase in viral incidence (percentage of plants infected as determined by RT‐PCR) in the mutant compared to *Nip* (Fig. 3D). Thus disruption of auxin receptor TIR1 increased rice susceptibility to RBSDV infection.

**Figure 4:**
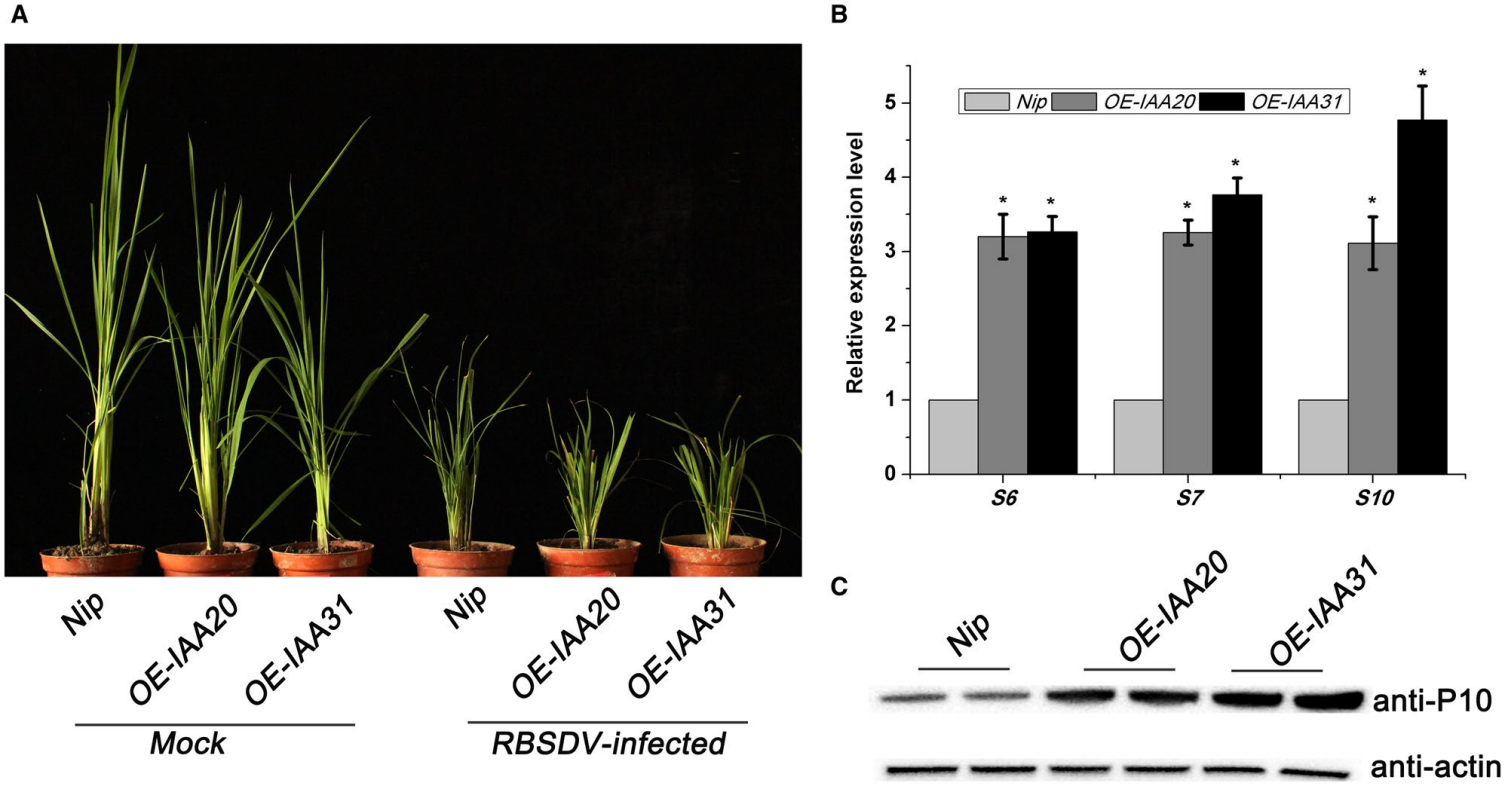
The effect of overexpression *OE‐IAA20* and *OE‐IAA31* on RBSDV infection. (A) The symptoms on mock‐inoculated or RBSDV‐infected *Nip* and mutant plants, respectively. The symptoms were observed at 30 days post‐inoculation. (B) The relative expression levels of RBSDV *S6*, *S7* and *S10* in RBSDV‐infected *Nip*, *OE‐IAA20* and *OE‐IAA31* transgenic plants assessed by RT‐qPCR. The average values from three biological replicates are shown. Error bars represent ±SD. *, *P* ≤ 0.05. (C) Western blot of leaf extracts to detect the levels of RBSDV in inoculated control (*Nip*) and *OE‐IAA20 and OE‐IAA31* plants. The RBSDV P10 outer capsid protein was detected using an anti‐P10 antibody. Actin antibody was used as an internal reference.

Aux/IAA proteins play important roles in repressing auxin signalling. In normal conditions, Aux/IAA proteins bind to the transcription factor ARFs, repressing the expression of auxin response genes. When the IAA concentration increases, the TIR1 receptor can bind Aux/IAA proteins to form a SCF^TIR1^/AFB–auxin–Aux/IAA complex leading to polyubiquitination and degradation of Aux/IAA proteins. Recently, Aux/IAA proteins have been shown to be involved in plant–virus interactions (Jin *et al.*, 2016; Padmanabhan *et al.*, 2005). Since RBSDV infection altered the expression of *OsIAA20* and *OsIAA31* (Fig. 1B), we then produced transgenic rice overexpressing these two genes driven by the *35S* promoter, with a 4XHA epitope tag at the N‐terminus (*OE‐IAA20* and *OE‐IAA31*). Western blot assays confirmed the expression of OsIAA20 and OsIAA31 in the corresponding transgenic rice plants (Fig. S2). The transgenic plants overexpressing *OE‐IAA20* and *OE‐IAA31* and wildtype *Nip* were then inoculated with RBSDV. Thirty days after inoculation, both transgenic plants had more severe dwarfing than the *Nip* controls (Fig. 4A). There were corresponding increases in RBSDV RNA levels as measured by RT‐qPCR (Fig. 4B) and in the expression levels of viral P10 as shown by western blot (Fig. 4C). These results indicate that repressing auxin signalling by overexpression of Aux/IAA proteins promotes rice susceptibility to RBSDV infection.

To exclude the possibility that the auxin signalling mutants had altered resistance to the insect vector, we estimated SBPH resistance as described previously (He *et al.*, 2017). Ten‐day‐old seedlings were infested with SBPH at seven insects per seedling, and insect survival was evaluated 3 and 5 days later. As shown in Fig. S3, there was no significant difference in SBPH mortality between the wildtype plants and the auxin signalling mutants. Together, the results indicate that auxin signalling plays an important role in plant defence against RBSDV.

### The involvement of the JA pathway in auxin‐mediated defence against RBSDV infection

**Figure 5:**
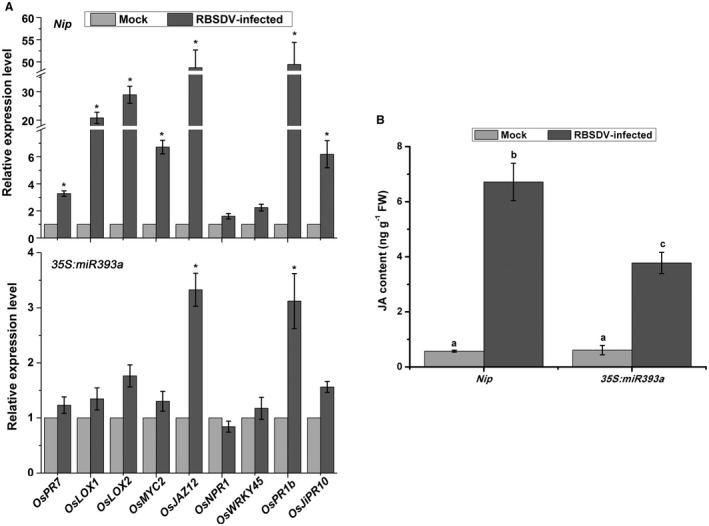
The effect of repressing auxin signalling on jasmonic acid (JA) or salicylic acid (SA) pathway genes in response to RBSDV infection. (A) The expression of JA pathway or SA pathway genes in the *Nip* controls (upper panel) or *35S:miR393a* mutant (bottom panel) in response to RBSDV infection. Data are shown as relative expression levels in virus‐infected plants in comparison to the control plants. *UBQ5* was used as the internal reference gene. Values are means ± SD of three biological replicates. Statistically significant differences from the control are indicated: *P ≤ 0.05. (B) The content of JA in *Nip* controls or *35S:miR393a* mutant in response to RBSDV infection. Values are means ± SD of three biological replicates. Different letters at the top of columns indicate significant differences at *P* ≤ 0.05 (*n* = 3).

Previous research has shown that auxin signalling interacts either positively or antagonistically with the SA or JA pathways in response to pathogen invasion (Qi *et al.*, 2012; Wang *et al.*, 2007). We therefore examined the expression of JA and SA pathway genes in our auxin signalling mutants following viral infection. As shown in Fig. 5A, the expression of JA biosynthesis‐related genes (*OsOPR7*, *OsLOXs*) and JA signalling‐related genes (*OsMYC2*, *OsJAZ12*) was markedly increased in RBSDV‐infected *Nip* plants compared with mock‐inoculated *Nip* plants (*P* ≤ 0.05) (Fig. 5A, upper panel), but there was little obvious difference in expression between RBSDV‐infected and mock‐inoculated *35S:miR393* mutant plants (Fig. 5A, bottom panel). In our previous study, the defence‐related *OsPR* genes (such as *OsPR1b*, *OsJiPR10*) were activated upon RBSDV infection (He *et al.*, 2017; Zhang *et al.*, 2019b). The expression of *OsPR1b* and *OsJiPR10* was also tested in RBSDV‐infected *35S:miR393* mutant and *Nip* plants. Consistent with our previous results, the expression of *OsPR1b* and *OsJiPR10* was remarkably enhanced in RBSDV‐infected *Nip* compared to non‐infected control plants (*P* ≤ 0.05) (Fig. 5A, upper panel). Interestingly, the expression of these genes in RBSDV‐infected *35S:miR393* mutant was slightly increased compared to the mock‐inoculated *35S:miR393* plants (Fig. 5A, bottom panel). Similar results were obtained using the *OE‐IAA20* and *OE‐IAA31* transgenic plants (Fig. S4). These results indicate that the JA pathway and defence‐related *OsPR* genes normally activated by RBSDV infection were repressed when auxin signalling was disrupted.

To confirm whether the JA content was affected by repressing auxin signalling, the JA concentration was measured in RBSDV‐infected rice plants at 40 dpi (Fig. 5B). Consistent with our previous results, the JA concentration was more than six‐fold greater in RBSDV‐infected *Nip* than in mock‐inoculated *Nip* plants, while its concentration in RBSDV‐infected *35S:miR393* mutants was only about three‐fold greater than that in the corresponding controls. Thus the induction of JA in response to RBSDV was impaired in the auxin signalling mutant.

The expression levels of SA signalling genes (*OsNPR1*, *OsWRKY45*) were also measured by RT‐qPCR in RBSDV‐infected plants. As shown in Fig. 5A, the expression of these genes did not appear to respond significantly to RBSDV in either the *35S:miR393* mutant or *Nip* plants. Similar results were obtained using the *OE‐IAA20* and *OE‐IAA31* mutants (Fig. S4). These results suggest that the SA pathway was not involved in the interaction between RSBDV and auxin signalling. Therefore, disruption of auxin signalling affected the activation of the JA pathway, but not the SA pathway, in response to RBSDV infection.

### The involvement of *OsRboh*‐mediated ROS levels in the interaction between auxin signalling and RBSDV infection

**Figure 6:**
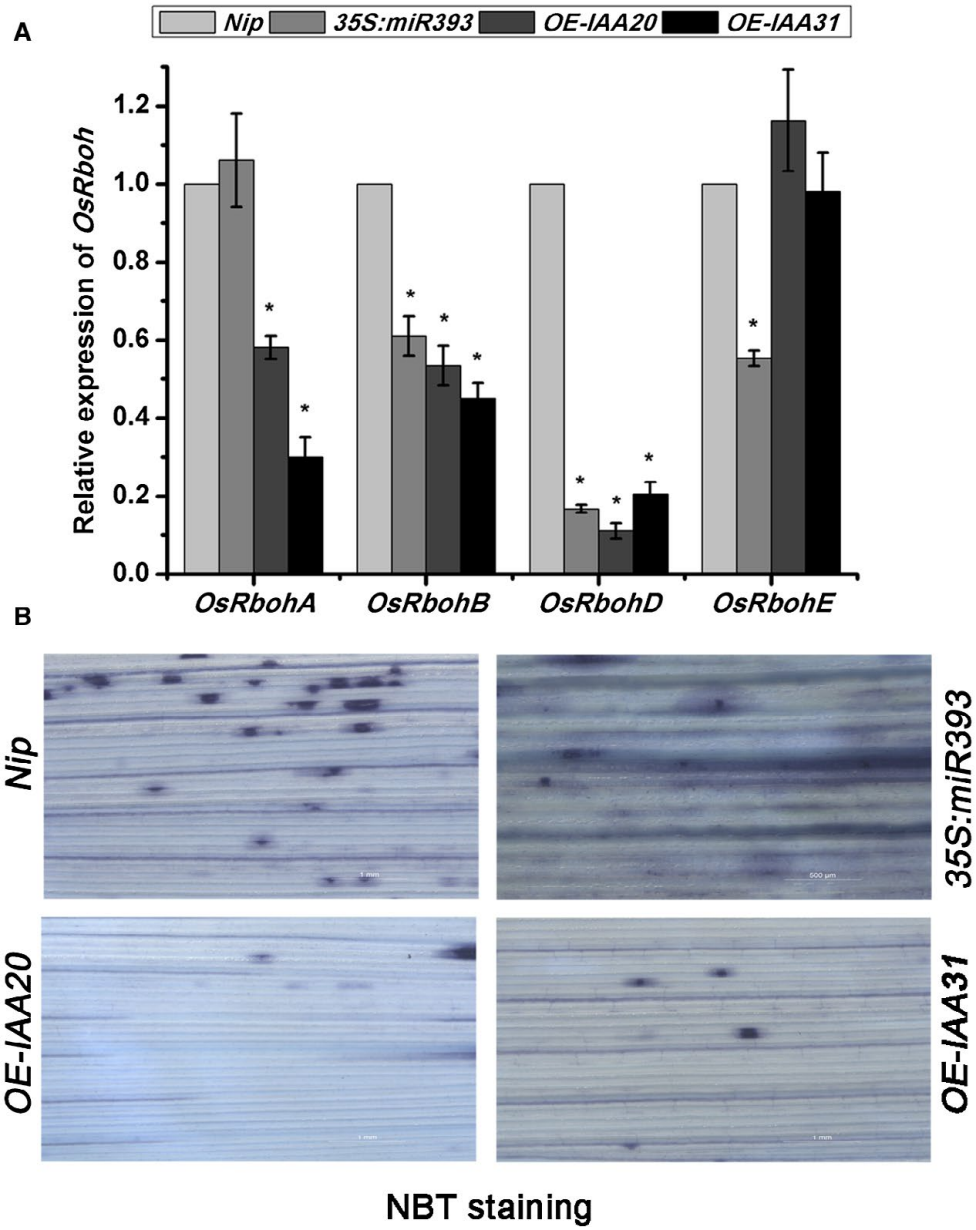
The effect of auxin signalling mutants on reactive oxygen species (ROS) homeostasis. (A) RT‐qPCR analysis of the expression of *OsRboh* genes in the seedlings of auxin signalling mutants compared with that in *Nip*. Data are shown as relative expression levels in the mutants in comparison to the wildtype *Nip*. *UBQ5* was used as the internal reference gene. Values are means ± SD of three biological replicates. Statistically significant differences from the control are indicated: *P ≤ 0.05. (B) *In situ* detection of leaf ROS levels in *Nip* or auxin signalling mutants using nitroblue tetrazolium staining.

It has recently been reported that accumulation of basal ROS plays an important role in antiviral immunity (Wu *et al.*, 2017; Xie *et al.*, 2018). In plants, NADPH oxidases (respiratory burst oxidase homologs, *Rboh*) play key roles in generating ROS by catalysing the conversion of O_2_ to superoxide (O_2_^‐^) (Suzuki *et al.*, 2011). The rice genome encodes nine *Rboh* proteins (A–I) (Wang *et al.*, 2013) of which *OsRbohA*, *OsRbohB* and *OsRbohE* are reported to be involved in ROS production during pathogen invasion (Wong *et al.*, 2007; Yoshie *et al.*, 2005). To verify the impact of auxin signalling on *OsRboh*‐mediated ROS levels, the expression of *OsRboh* transcripts was monitored in auxin signalling mutants. As shown in Fig. 6A, the expression of *OsRbohB* and *OsRbohD* was significantly decreased in all auxin signalling mutants compared to the wildtype *Nip* (*P* ≤ 0.05). In addition, the expression of *OsRbohA* in the *OE‐IAA20* and *OE‐IAA31* transgenic plants and of *OsRbohE* in the *35S:miR393* mutant were all significantly decreased (*P* ≤ 0.05). The ROS levels as measured by nitroblue tetrazolium (NBT) staining were obviously less in the auxin signalling mutants than in the *Nip* controls (Fig. 6B). Thus, the expression of *OsRboh* and basal ROS levels was affected by disrupting auxin signalling.

**Figure 7:**
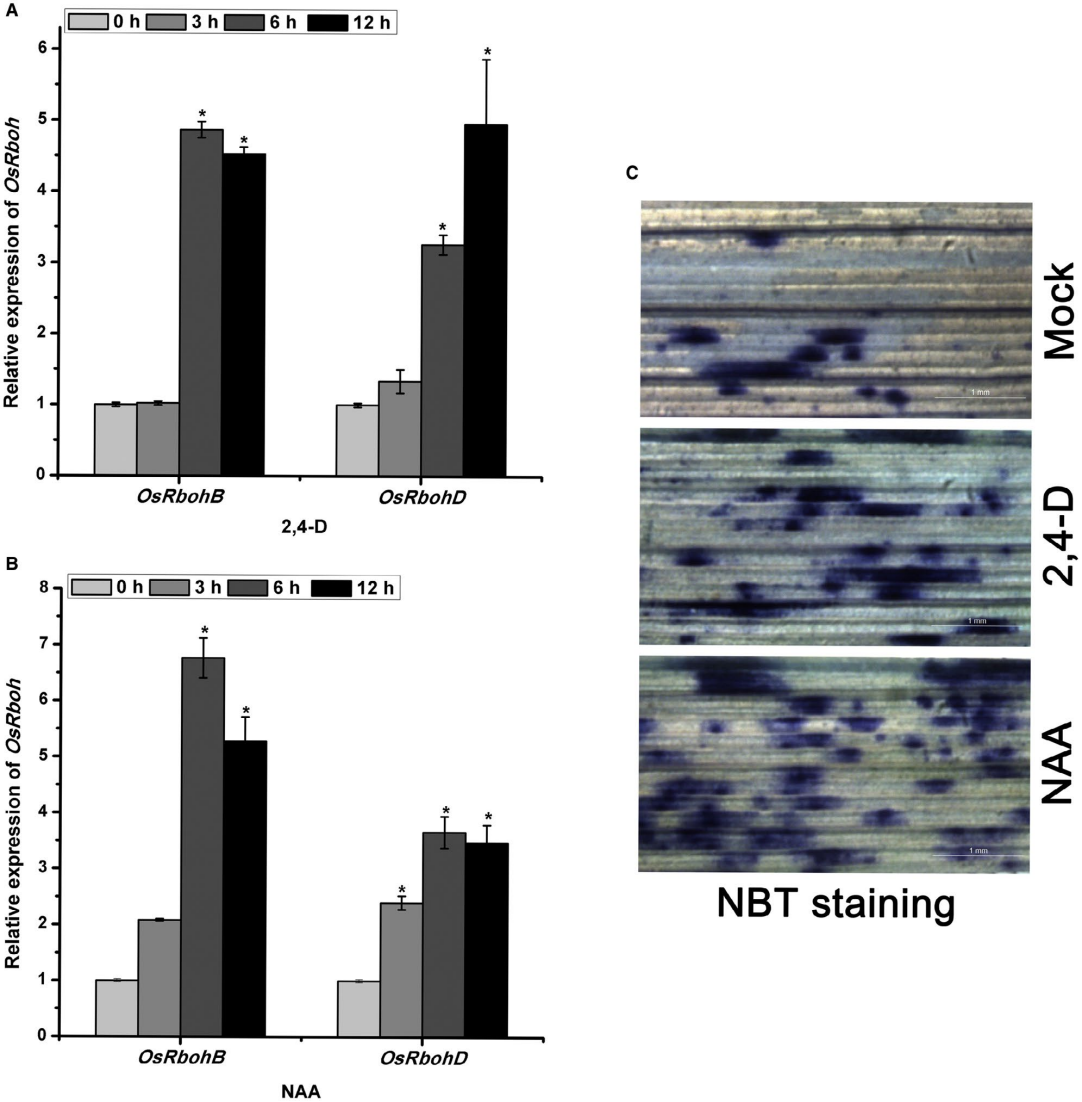
The effect of synthetic auxin treatments on OsRboh‐mediated ROS levels. (A) and (B) RT‐qPCR verification of relative expression of *OsRbohB* and *OsRbohD* genes in rice seedlings treated with 1 μM 2,4‐D (A) or 1 μM NAA (B) compared with 0.1% Triton X‐100 treated rice controls. Treatments used 10‐day‐old rice seedlings. Each sample contained a pool of eight to ten plants and was collected 0, 3, 6 or 12 h after spraying. The average values from three biological replicates are shown. * at the top of columns indicates significant difference at *P* ≤ 0.05. (C) Nitroblue tetrazolium (NBT) staining of rice leaves treated with 0.1% Triton X‐100 (mock), 1 μM 2,4‐D or 1 μM NAA.

To further elucidate the interaction between the auxin pathway and the *OsRboh*‐mediated ROS levels, 10‐day‐old rice seedlings were treated with the synthetic auxins 2,4‐dichlorophenoxyacetic (2,4‐D) or 1‐naphthylacetic acid (NAA). The expression of *OsRbohB* and *OsRbohD* was significantly increased after treatment with 1 μM 2,4‐D or 1 μM NAA (*P* ≤ 0.05) (Fig. 7A,B), and ROS levels, as shown by NBT staining, were also greater than in the controls treated with 0.1% Triton X‐100 (Fig. 7C). These results show that the auxin pathway positively modulated the *OsRboh*‐mediated ROS levels. Since increased ROS levels enhance resistance to RBSDV, we conclude that *OsRboh*‐mediated accumulation of ROS is at least partly responsible for the auxin signalling‐regulated plant defence against RBSDV.

**Figure 8:**
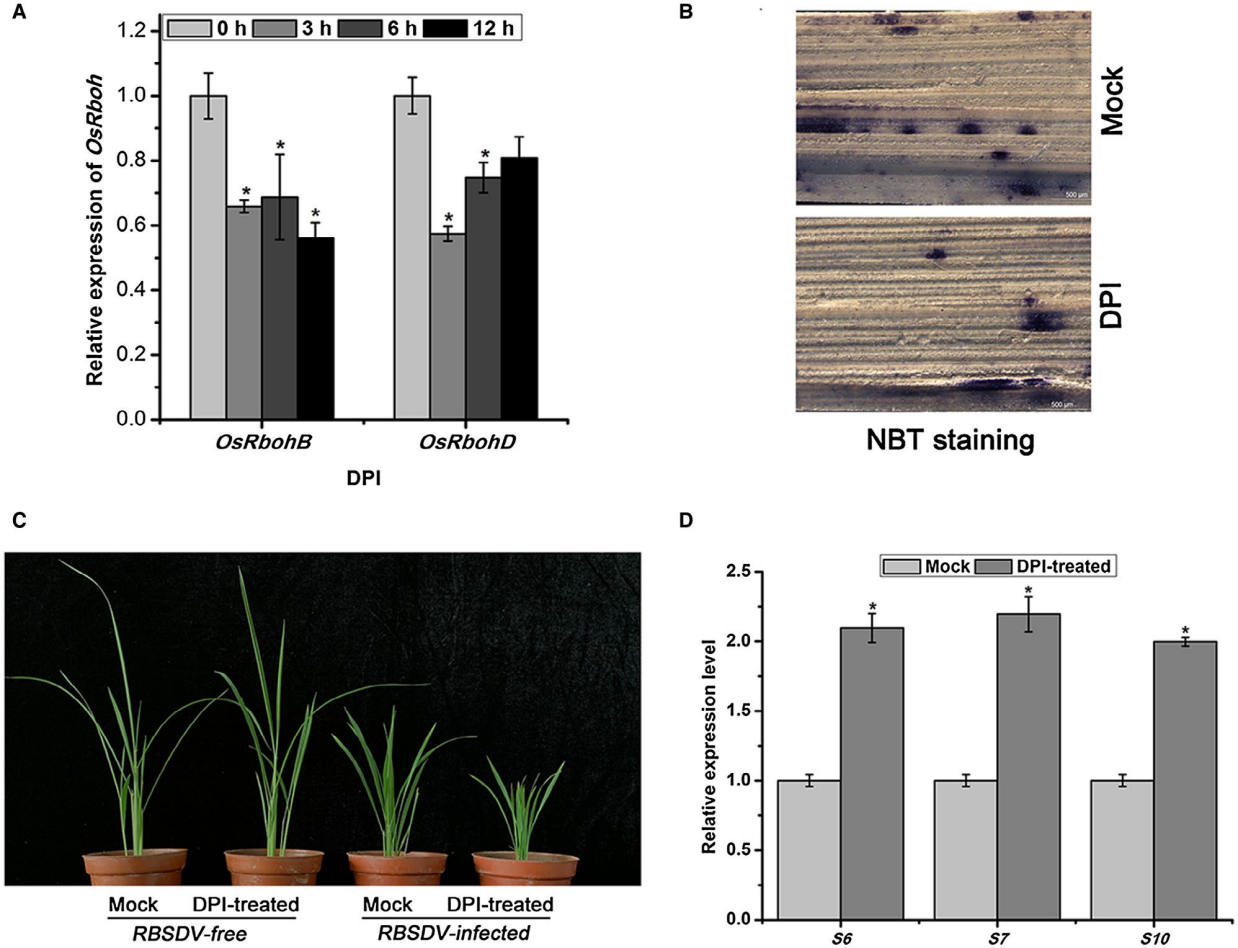
The effect of OsRboh‐mediated reactive oxygen species on RBSDV infection. (A) The effect of treatment with 100 μM diphenyliodide (DPI) on the expression of *OsRboh*. Each result represents not less than three biological repeats. * at the top of columns indicates significant difference from the mock‐treated control at *P* < 0.05. (B) Nitroblue tetrazolium (NBT) staining of rice seedlings treated with 100 μM DPI. (C) The effect of treatment with 100 μM DPI on the symptoms of RBSDV at 30 days post‐inoculation. (D) The effect of treatment with 100 μM DPI on the relative expression levels of RBSDV S6, S7 and S10 in RBSDV‐infected plants assessed by RT‐qPCR. Each treatment consisted of three biological replicates of at least 30 seedlings each. Different letters at the top of columns indicate significant differences at *P* < 0.05 (*n* ≥ 3) by Fisher's least significant difference tests.

To confirm the effect of *OsRboh*‐mediated ROS accumulation on RBSDV infection, diphenyliodide (DPI)‐treated rice plants were inoculated with RBSDV. DPI is a well‐known inhibitor of RBOH activity (Hyodo *et al.*, 2017; Lv *et al.*, 2018). As shown in Fig. 8A, the expression of *OsRbohB* and *OsRbohD* in DPI‐treated rice seedlings was significantly reduced compared with that in untreated controls (*P* ≤ 0.05). The ROS levels as measured by NBT staining were much lower in the DPI‐treated rice seedlings than in mock‐treated plants (Fig. 8B). When these DPI‐treated rice plants were inoculated with RBSDV, viral symptoms at 30 dpi were more severe in DPI‐treated plants than in mock‐treated plants (Fig. 8C). An RT‐qPCR assay indicated that the RBSDV RNA levels (S6, S7 and S10) were about two‐fold greater in DPI‐treated plants than in the untreated controls (*P* ≤ 0.05) (Fig. 8D). These results demonstrate that *OsRboh*‐mediated ROS accumulation plays an important role in rice defence against RBSDV infection.

## Discussion

Previous studies have mainly focused on the influence of viral infection on plant auxin homeostasis and signalling. For example, *Tobacco mosaic virus* (TMV) p126 protein was shown to interact with several Aux/IAA proteins to affect the expression of a large number of auxin‐responsive genes (Padmanabhan *et al.*, 2005, 2008). *Rice dwarf virus* (RDV) P2 protein interfered with auxin signalling by directly interacting with and stabilizing the OsIAA10 protein to enhance viral infection and disease development (Jin *et al.*, 2016). However, the role of auxin signalling in the antiviral mechanism remains unclear. Here, we have shown that auxin signalling plays an important role against RBSDV. The induction of the JA pathway in response to RBSDV was undermined in auxin signalling mutants. These results suggest that auxin signalling is required to activate the JA pathway upon viral infection. Recent studies showed that auxin defective mutants of *Arabidopsis* were more susceptible than wild‐type plants to a necrotrophic pathogen (Qi *et al.*, 2012). Application of IAA and methyl jasmonate together synergistically induced the expression of defence‐related genes *PDF1.2* and *HEL*. Those results indicated that auxin positively interacted with the JA pathway against necrotrophic pathogens. Moreover, JA was shown to induce the auxin biosynthesis genes *ASA* and *YUCCA*. However, the influence of auxin signalling on the JA pathway during pathogen invasion has been little reported. Here, our results suggest that auxin plays important roles in the activation of the JA pathway, and that induction of the JA pathway may be part of the auxin signalling‐mediated defence against RBSDV.

ROS were initially considered to be harmful to cells, but are now known to act as signalling molecules to regulate cell growth and defence (Mittler *et al.*, 2011). The crosstalk between auxin and ROS has mainly been reported from studies of plant growth and development (Joo *et al.*, 2001, 2005). A recent report showed that auxin‐induced ROS production was important for polar root hair growth by inducing the expression of ROS‐generating enzymes (NADPH oxidases) (Mangano *et al.*, 2017). Auxin signalling could activate RSL4, which directly targets the NADPH oxidases through auxin response factors to modulate ROS levels. However, the interplay between auxin and ROS has been little reported in the context of plant defence. Recently, ROS were demonstrated to be involved in plant–virus interactions (Hyodo *et al.*, 2017; Wu *et al.*, 2017; Xie *et al.*, 2018). Two positive‐strand RNA viruses, *Red clover necrotic mosaic virus* (RCNMV) and *Brome mosaic virus*, were shown to hijack the host's ROS‐generating machinery and trigger intracellular ROS bursts (Hyodo *et al.*, 2017). These bursts are required for viral RNA replication. However, ROS were shown to play vital roles in plant defence against rice viruses (Wu *et al.*, 2017). Our previous results also showed that high basal ROS levels can enhance antiviral defence against RBSDV infection (Xie *et al.*, 2018). Here, our results indicate that auxin can induce the expression of *OsRboh* and that disruption of auxin signalling decreased the expression of *OsRboh*. Repressing the expression of *OsRboh* increased rice susceptibility to RBSDV infection. These results indicate that ROS reduction by repression of auxin signalling contributes in part to rice susceptibility to RBSDV.

In summary, our results showed that RBSDV infection affected auxin homeostasis and down‐regulated the expression of auxin pathway genes. Repressing auxin signalling uncovered the positive roles of auxin signalling in RBSDV infection. Further research revealed that activation of JA‐mediated defence and accumulation of *OsRboh*‐mediated ROS played vital roles in the interaction between auxin signalling and RBSDV infection. Our results confirm that auxin signalling plays a positive role in rice defence against RBSDV, but more research is needed to determine the mechanism by which RBSDV represses the auxin pathway.

## Experimental Procedures

### Plant materials and growth conditions

Rice seeds of Wuyujing No.3 and Huaidao No. 5 were used for this study. The cultivar rice *Nipponbare* (*O. sativa, NIP*) was used as the wild‐type control. The auxin signalling mutant *35S:miR393* was obtained from Dr Hongwu Bian (Zhejiang University) (Bian *et al.*, 2012). The *DR5:GUS* transgenic plants were gifts from Dr Yanhua Qi (Zhejiang University). RBSDV‐infected plants collected from fields (Shandong Province, China) were used as a virus source. Rice plants were grown in the greenhouse with14/10 h light/dark cycle at 28–30 °C.

### Construction and rice transformation of overexpression vector

To prepare constructs to overexpress the *OsIAA20 and OsIAA31* genes, the respective open reading frames were first amplified from cDNA of *NIP* using primers incorporating *Bam*HI and *Sac*I sites (Table S1). The pCV1300 vector was digested with *Bam*HI and *Sac*I, which contains the cauliflower mosaic virus (CaMV) doubled 35S promoter to drive transcription. The *OsIAA20* and *OsIAA31* PCR products were then combined into the pCV1300 vector (Sun *et al.*, 2013a,b) to create the respective recombinant vectors OE‐IAA20 and OE‐IAA31. These vectors were introduced into *Agrobacterium* (strain GV3101) using electroporation and transformed into *NIP* rice as described previously (Zhang *et al.*, 2019a).

### RBSDV inoculation assay

The detailed assays were conducted as described previously (Zhang *et al.*, 2019a). Non‐viruliferous SBPH were reared on Wuyujing No. 3 seedlings at 26 °C in a room under artificial light. Briefly, to acquire virus, large numbers of first‐ or second‐instar SBPH were prepared and then released onto RBSDV‐infected rice plants. After feeding for 3–5 days, instar nymphs were transferred to healthy Wuyujing No. 3 seedlings for 10–12 days (an incubation period). For RBSDV inoculation, planthoppers were transferred to 10‐day‐old rice seedlings (two to three leaf stage) to feed for 3 days (about three SBPH on each of 35–40 seedlings per treatment). The rice seedlings were then grown in the greenhouse to develop symptoms. RBSDV‐infected plants were stunted and had darkened leaves. Plant height was measured and RT‐PCR using virus‐specific primers (Table S1) was used to confirm infection and determine the percentage of plants infected (viral incidence). Simultaneously, to detect any effect of mutant rice plants on insect mortality, the rice seedlings were infested with SBPH at seven insects per seedling and their survival was measured 3 and 5 days later. The inoculation experiments had at least three biological repeats.

### Foliar application of chemicals

Ten‐day‐old Huaidao No. 5 seedlings were grown in glass beakers (35–40 seedlings per beaker) and sprayed with 25 mL 1 μM 2,4‐dichlorophenoxyacetic acid (2,4‐D, Sigma‐Aldrich), 1 μM 1‐naphthylacetic acid (NAA, Sigma‐Aldrich, St Louis, MO, USA) or 100 μM DPI (MedChemExpress, Monmouth Junction, NJ, USA). Solutions also contained 0.1% Triton X‐100 and control plants were sprayed with 25 ml 0.1% Triton X‐100. For RT‐qPCR, samples of eight to ten plants per beaker were collected and pooled 0, 3, 6 or 12 h after spraying. Each experiment used not less than three biological repeats.

### NBT staining assay

Superoxide was detected with nitroblue tetrazolium (NBT). The detailed procedures were based on those described previously (Xia *et al.*, 2009; Zhang *et al.*, 2019a). Thirty‐day‐old rice leaves were cut into 1–2 cm pieces and infiltrated with 2 mL NBT solution. The solution contained 6 mM NBT (Sigma) and 10 mM sodium citrate (pH = 6.0). The leaves were vacuum infiltrated (10 min, 60 kPa pressure) and incubated at 37 °C for 2 h. Then the rice leaves were rinsed in 75% ethanol twice for 30 min each and finally rinsed with absolute ethanol to bleach out the chlorophyll. The samples were then stored in 20% glycerol and photographed under a stereomicroscope.

### Analysis of IAA and JA concentrations

RBSDV‐infected and mock‐inoculated rice leaves were collected at 40 dpi and powdered in liquid nitrogen. The phytohormones were extracted and analysed as described previously with slight modifications (Fu *et al.*, 2012; He *et al.*, 2017). To measure IAA concentrations, an internal standard ^2^H_2_‐IAA (100 pmol) was added to a 20 mg sample. Samples were purified through a column purification and examined by ultra‐high‐performance liquid chromatography–triple quadrupole mass spectrometry (UPLC‐MS/MS) with negative electrospray ionization. To quantify JA, 2 mL of methanol containing ^2^H_5_‐JA (95 pmol) was added to 200 mg of leaf powder, and then purified by an Oasis mode anion exchange (MAX) solid phase extraction (SPE) column. The supernatants were collected and then analysed with UPLC‐MS/MS. Each sample consisted of at least four to five pooled plants and was replicated at least three times.

### RNA extraction and RT‐qPCR

To analyse gene expression levels, total RNA was isolated from rice leaves of 30‐day‐old plants using TRIzol reagent (Invitrogen, Carlsbad, CA, USA) following the manufacturer's protocol. cDNA was synthesised using the Tiangen fast quant RT kit (Tiangen, Beijing, China). The RT‐qPCR assay was performed using the ABI7900HT Sequence Detection System (Applied Biosystems, CA, USA) with ChamQTM SYBR qPCR Master Mix (Low ROX Premixed). *OsUBQ5* (AK061988) was used as an internal control (Sun *et al.*, 2015). The relative expression levels of genes were determined using the 2^‐ΔΔC (t)^ method. Each treatment had at least three biological replicates of three to five plants each and there were three technical replicates per sample. The primers used for RT‐qPCR are listed in Table S1.

### Protein Analysis

To detect the OsIAA20 and OsIAA31 proteins in the transgenic *OE‐IAA20* and *OE‐IAA31* rice plants, western blot analysis was performed using HA‐tag antibody (TransGen Biotech, Beijing, China). Western blots to test viral accumulation in RBSDV‐infected plants used an anti‐P10 antibody to detect the RBSDV P10 outer capsid protein (Lu *et al.*, 2019). Fresh leaf samples were ground in liquid nitrogen and extracted as described previously (Zhang *et al.*, 2019b). Briefly, 0.1 g samples were extracted in 300 μL SDS lysis buffer (100 mM Tris‐HCl, pH = 6.8, 10% SDS) and boiled with 5 × protein loading buffer for 10 min. The proteins were separated by 10–12% sodium dodecyl sulphate polyacrylamide gel electrophoresis, and then transferred to a polyvinylidene fluoride membrane (previously activated with methanol for 15 s). The membrane was blocked with 10% non‐fat dry milk diluted in tris buffered saline tween for 2 h at 25 °C, and then the primary antibody (1:5000) in blocking buffer was added for 2 h. The secondary antibody in blocking buffer was then added (1:10 000) and incubated for 1.5 h. Actin (Abbkine, A01050‐3) antibody was used as a reference protein. The proteins were detected using an ECL kit (Pierce, Rockford, USA) in the BIO‐RAD ChemiDoc MP Imaging System (Bio‐Rad, CA, USA).

### GUS staining assays

GUS staining assays were done as described previously (Shen *et al.*, 2013). Briefly, the rice leaves were cut into 10 cm pieces and infiltrated with 15 mL GUS staining solution using a brief vacuum infiltration. The solution included 0.5 mM X‐gluc (Sigma), 100 mM phosphate buffer (NaH_2_PO_4_·2H_2_O, Na_2_HPO_4_·12H_2_O, pH = 7.0), 1 mM K_3_[Fe(CN)_6_], 1 mM K_4_[Fe(CN)_6_], 10 mM Na_2_EDTA, 20%(v/v) methanol and 0.5% (v/v) Triton X‐100. After incubation at 37 °C for 1–2 h, the leaf pieces were washed with 75% ethanol twice for 30 min each time and finally rinsed with absolute ethanol before microscopic examination to determine GUS activity.

### Statistical analysis

Data were analysed using one‐way or two‐way ANOVA with Fisher's least significant difference tests. A *P*‐value ≤ 0.05 was considered statistically significant. All analyses were performed using ORIGIN 8.0 software.

## Competing Interests

The authors declare that they have no competing interests.

## Supporting information


**Fig. S1** The *cis*‐element in the promoters of *OsIAA20* and *OsIAA31*.

**Fig. S2** Western blot of leaf extracts to confirm the overexpression of *OE‐IAA20* and *OE‐IAA31* in the respective transgenic plants compared to the Nip control.

**Fig. S3** The mortality of small brown planthoppers on control (*Nip*) and auxin signalling mutant plants.

**Fig. S4** The expression levels of jasmonic acid (JA) or salicylic acid (SA) pathway genes in *OE‐IAA20* (upper panel) and *OE‐IAA31* (lower panel) mutants in response to RSBDV.

**Table S1** The primers used in this study.
