# Supplementary material for: Suppression of auxin signalling promotes rice susceptibility to Rice black streaked dwarf virus infection
Source: Mol Plant Pathol. 2019 Jun 27;20(8):1093–104. doi: 10.1111/mpp.12814 (PMC6640184; doi:10.1111/mpp.12814)
Supplement: Supplementary file 1 — Fig. S1 The cis‐element in the promoters of OsIAA20 and OsIAA31. [file MPP-20-1093-s001.pdf]

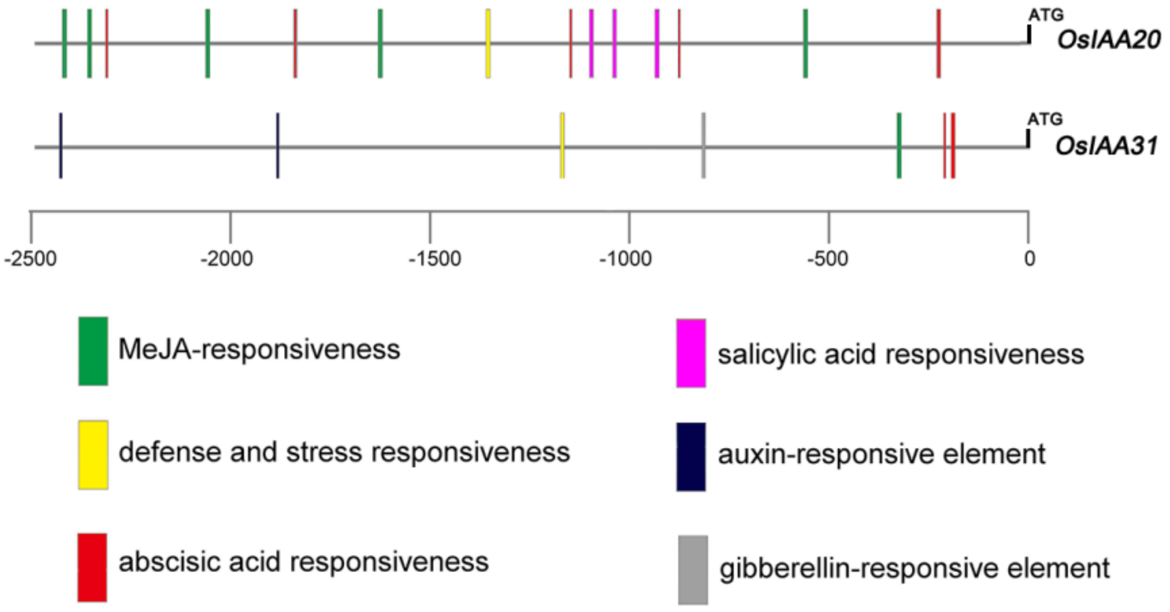

Fig. S1 The *cis*-element in the promoters of *OsIAA20* and *OsIAA31*. The 2.5 kb promoter sequence of *OsIAA20* and *OsIAA31* were analysed by PLANTCARE program. The different colours indicated the different binding element.
