# Supplementary material for: Suppression of auxin signalling promotes rice susceptibility to Rice black streaked dwarf virus infection
Source: Mol Plant Pathol. 2019 Jun 27;20(8):1093–104. doi: 10.1111/mpp.12814 (PMC6640184; doi:10.1111/mpp.12814)
Supplement: Supplementary file 2 — Fig. S2 Western blot of leaf extracts to confirm the overexpression of OE‐IAA20 and OE‐IAA31 in the respective transgenic plants compared to the Nip control. [file MPP-20-1093-s002.pdf]

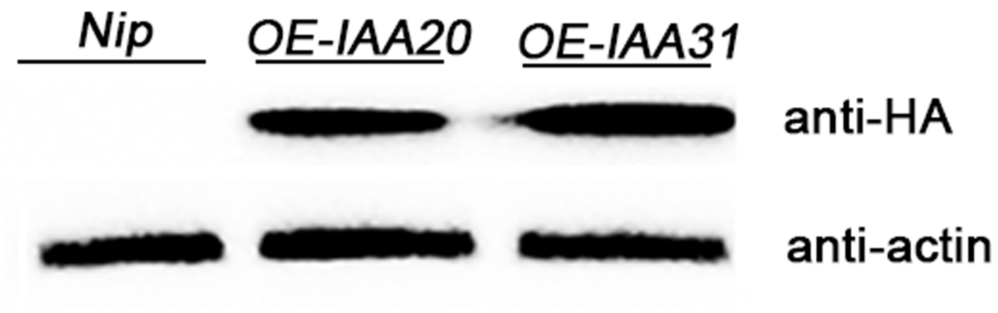

Fig. S2 Western blot of leaf extracts to confirm the overexpression of *OE-IAA20* and *OE-IAA31* in the respective transgenic plants compared to the *Nip* control. Proteins were detected using the HA antibody. Actin antibody was used as an internal reference.
