# Supplementary material for: Suppression of auxin signalling promotes rice susceptibility to Rice black streaked dwarf virus infection
Source: Mol Plant Pathol. 2019 Jun 27;20(8):1093–104. doi: 10.1111/mpp.12814 (PMC6640184; doi:10.1111/mpp.12814)
Supplement: Supplementary file 3 — Fig. S3 The mortality of small brown planthoppers on control (Nip) and auxin signalling mutant plants. [file MPP-20-1093-s003.pdf]

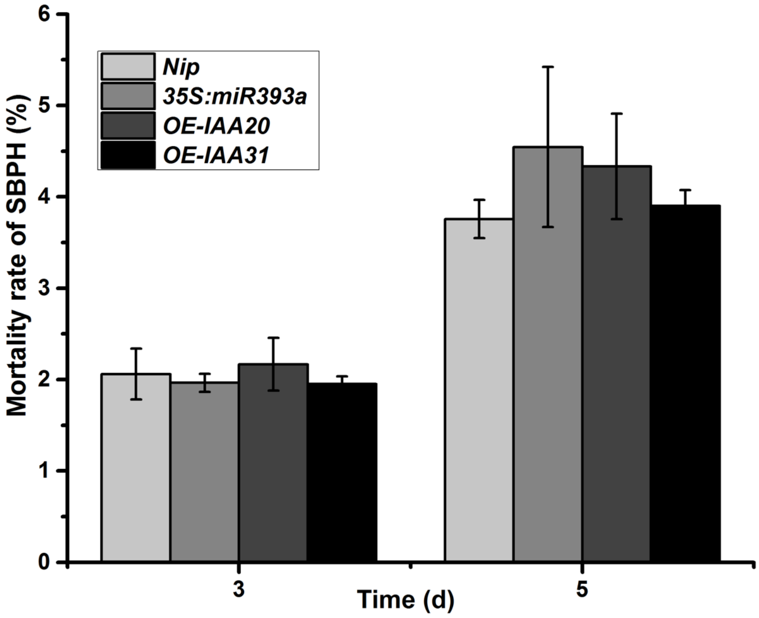

Fig. S3 The mortality of small brown planthoppers on control (*Nip*) and auxin signalling mutant plants. Ten-day-old seedlings were infested with SBPH at 7 insects per seedling and insect survival was measured 3 and 5 d later. Each treatment used at least 20 seedlings. Data are means ± SD from three biological replicates.
