# Supplementary material for: Suppression of auxin signalling promotes rice susceptibility to Rice black streaked dwarf virus infection
Source: Mol Plant Pathol. 2019 Jun 27;20(8):1093–104. doi: 10.1111/mpp.12814 (PMC6640184; doi:10.1111/mpp.12814)
Supplement: Supplementary file 4 — Fig. S4 The expression levels of jasmonic acid (JA) or salicylic acid (SA) pathway genes in OE‐IAA20 (upper panel) and OE‐IAA31 (lower panel) mutants in response to RSBDV. [file MPP-20-1093-s004.pdf]

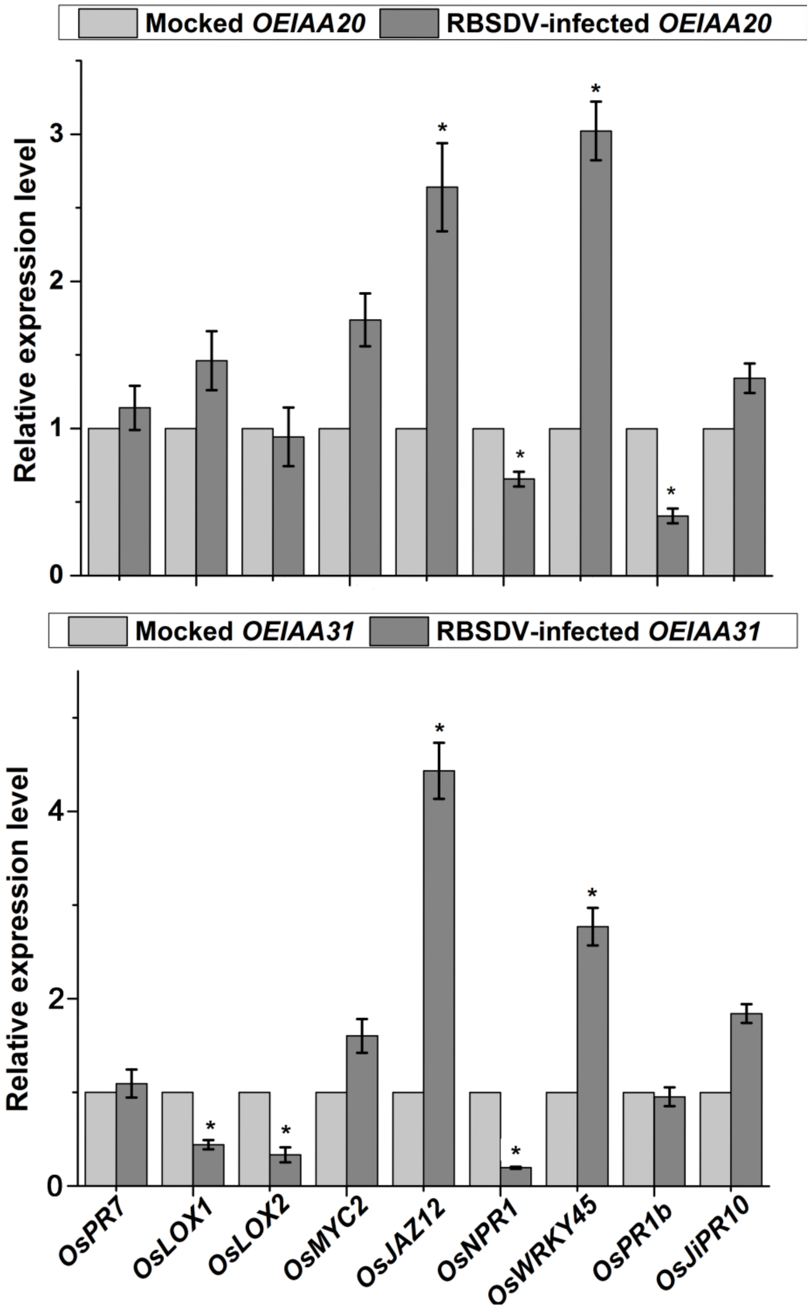

Fig. S4 The expression levels of jasmonic acid (JA) or salicylic acid (SA) pathway genes in *OE-IAA20* (upper panel) and *OE-IAA31* (lower panel) mutants in response to RSBDV. Data are shown as relative expression levels of virus-infected plants in comparison to the control plants. UBQ5 was used as the internal reference gene. Values are means ±SD of three biological replicates. Statistically significant differences from the control are indicated: *, $p \leq 0.01$.
