# Supplementary material for: Suppression of auxin signalling promotes rice susceptibility to Rice black streaked dwarf virus infection
Source: Mol Plant Pathol. 2019 Jun 27;20(8):1093–104. doi: 10.1111/mpp.12814 (PMC6640184; doi:10.1111/mpp.12814)
Supplement: Supplementary file 5 — Table S1 The primers used in this study. [file MPP-20-1093-s005.pdf]

Additional file 4 the primers used in this study

For RT-PCR		
RBSDV	RB-F	AGCACTACAGATGTTACACAC
	RB-R	GCCTGTCCCTATCAGATTTCAAC
For RT-qPCR		
IAA genes	qOsPINL1-F	TAGCGAGCGTAATCGTCGTC
	qOsPINL1-R	TGCGTGATGGTCCCAATGTT
	qOsPIN1-F	AGTCTCGGTCTGTTCATGGC
	qOsPIN1-R	ACTCCTTGGCGAAGACGAAG
	qOsPINL2-F	GGGGGAATCTGACACAAGGG
	qOsPINL2-R	AATCCAACCCCATAGGCAGC
	qOsPIN6-F	CATGTCCAAGTCAGGCACAG
	qOsPIN6-R	GAGGTAGGGCTGCCTGTATG
	qOsYUCCA6-F	CATACTGGCCACCGGATACC
	qOsYUCCA6-R	GCCAACAGAGTAGAGCCCTG
	qOsYUCCA1-F	GTTGGGACGCTAGACCACAT
	qOsYUCCA1-R	AAGTCACCGGCATCCTTGAG
	qOsYUCCA-F	AGTGGCTCAAGAGCGATGAC
	qOsYUCCA-R	CATCTGCTTGCTGATGTCGT
	qOsTAA2-F	TGACGAAGTTTGTGGAGCTG
	qOsTAA2-R	CGAAGAGGAGTGGTCATCGT
	qOsTAA4-F	CACGGCAAGGAGGACATCTT
	qOsTAA4-R	TCCTCCCACTCGCATTTCAC
	qOsIAA20-F	GCTTGACCTTCTCAACAGCG
	qOsIAA20-R	CTCTTGTTGCCCATTTCGCC
	qOsIAA31-F	CAAGGTGTACAAGGGCTACC
	qOsIAA31-R	CATGATCCTCAACCTCTTGC
	qOsIAA7-F	CTCACTGCTGGAGCAAAGGA
	qOsIAA7-R	AACAAACATCCCCCATGGCA
	qOsGH3.2-F	TACATCCCCACGCTCAAGTT
	qOsGH3.2-R	GCTCGAAGTAGCCCATGTTG
	qOsGH3.8-F	TGGGCTACTTCGAGTTCCTC
	qOsGH3.8-R	GATGGAGAGGAGCACGTTCT
RBSDV	qRB-S6-F	AGCGTGTTGAAAACGAGATC
	qRB-S6-R	CGCTTTGCAAATTCAACAAG
	qRB-S7-F	CTCCCCGTAAGCCAAGTTT
	qRB-S7-R	AGGCAACTCAGTAGGAGCAG
	qRB-S10-F	AACAACCGACCAACAATCAC
	qRB-S10-R	GAGCAGGAACTTCACGACAG
defense related genes	qOsOPR7-F	TTGGGTTGGAAGCAGTGGAG
	qOsOPR7-R	GGATCGGGCGTGTAGAATGT
	qOsLOX1-F	TCCACCGACGAGGAGTACC
	qOsLOX1-R	TATCCTTGTTCCGGCCATCG
	qOsLOX2-F	CCACCGACGAGGAGTACCT
	qOsLOX2-R	CGCACCGATTCTTGAGCTTC
	qOsMYC2-F	AGCTCAACCAGCGCTTCTAC
	qOsMYC2-R	CCTTCTTGAGCGACTCCATC
	qOsJAZ12-F	TCTTCTACGACGGGAGGATG
	qOsJAZ12-R	TTGTCGTGATCCTGTGCTTC
	qOsNPR1-F	CACGCCTAAGCCTCGGATTA
	qOsNPR1-R	TCAGTGAGCAGCATCCTGACT
	qOsWRKY45-F	TTCCTTGTTGATGTGTCGTCTCA
	qOsWRKY45-R	CCCCCAGCTCATAATCAAGAAC
	qOsPR1b-F	GCGAGAAGAGCGACTACGAC
	qOsPR1b-R	GCCGGCTTATAGTTGCATGT
	qOsJiPR10-F	GACAAGTGCGAGTGCAAGTC
	qOsJiPR10-R	AGGGACTCCTTAGCCTTGGT
OsRboh	qOsRbohA-F	GCTTCCAAGGCCATTTGACA
	qOsRbohA-R	TGCCAGACCTGAAGAACCTT
	qOsRbohB-F	GGTTCCGACGTAACAAGCTC
	qOsRbohB-R	AACAGGAACAGCGAGGTACA
	qOsRbohD-F	CCTTGCTGGTTTCAATGCCT
	qOsRbohD-R	GGTCACCCCATAACTGCTCT
	qOsRbohE-F	ATGGGATGCTCACGGAAGAT
	qOsRbohE-R	TGCCAGATCTCGATGTACCC
For construction of overexpression vector	OE-IAA20-F	GTTCCAGATTACGCTGGATCCATGGAGCTCGAGCTCGGCCTC
	OE-IAA20-R	ATCGGGGAAATTCGAGCTCTTACACCAGTATCTTGAGCCGTT
	OE-IAA31-F	GTTCCAGATTACGCTGGATCCATGGAGAATCTGAAGGCGAC
	OE-IAA31-R	ATCGGGGAAATTCGAGCTCTTAACCCCTTGTTGCTCCTAGGC
